# Regional differences in amplitude and spatial homogeneity of muscle activity in the biceps femoris long head

**DOI:** 10.1007/s00421-025-05783-5

**Published:** 2025-04-17

**Authors:** Alexander Fassbender, Kiros Karamanidis, Wolfgang Potthast

**Affiliations:** 1 Institute of Biomechanics and Orthopaedics, German Sport University Cologne, Cologne, Germany; 2 School of Applied and Health Sciences, London South Bank University, London, UK; 3 Department of Sport Science, Faculty of Mathematics and Natural Sciences, University of Koblenz, Koblenz, Germany

**Keywords:** Biceps femoris long head, Hamstring injuries, Muscle activation, Modified entropy, High-density surface EMG

## Abstract

**Purpose:**

Hamstring injuries, particularly in the proximal Biceps femoris long head (BFlh), remain frequent in sports involving sprints and accelerations despite extensive research. Non-uniform muscle activity may contribute to these injuries by causing uneven load distribution. This study examines spatial homogeneity of muscle activity and amplitude in the proximal and distal BFlh at different knee flexion torque levels and muscle-tendon unit (MTU) lengths under controlled isometric conditions.

**Methods:**

Fifteen male recreational athletes performed unilateral isometric knee flexion contractions at three MTU lengths (0°, 45°, 90° hip flexion) and torque levels (30% _MVC90_, 60% _MVC90_, 90%_MVC90_) with high-density surface electromyography (HDsEMG) assessing proximal and distal activity.

**Results:**

The proximal BFlh exhibited lower spatial homogeneity and amplitude compared to the distal region across all conditions, with the largest homogeneity differences at lower torques and longer MTU lengths. Proximal homogeneity increased with torque and decreased with MTU length, while the distal region remained consistent. Amplitudes were lower proximally and decreased with MTU length in both regions.

**Conclusion:**

The proximal–distal differences in spatial homogeneity and amplitude within the BFlh reflect non-uniform activation patterns along the BFlh and the proximal regions lower spatial homogeneity and amplitude of activation reflect non-uniform patterns, possibly contributing to injury risk.

## Introduction

Field sports involve frequent sprints, accelerations, and directional changes, making lower extremity injuries the most common (Maniar et al. 2023). Hamstring injuries are particularly prevalent, now accounting for up to 24% of all injuries in European men’s professional football, with relative incidence and recovery times doubling over the past two decades despite advances in sports medicine (Ekstrand et al. 2023).

Hamstring injuries often occur during high-velocity sprints or accelerations, with the proximal M. Biceps femoris long head (BFlh) most frequently affected (Gronwald et al. 2022; Crema et al. 2016). Literature suggests that the interplay between multiple factors, including biomechanical, neuromuscular, and architectural factors, contributes to their occurrence (Huygaerts et al. 2020, Opar et al. 2022).

Results from biomechanical studies indicate that the late swing phase of the running cycle is the most demanding and the phase where the BFlh is most prone to injury (Chumanov et al. 2007; Kenneally‐Dabrowski et al. 2019; Danielsson et al. 2020). During this phase, peak eccentric stretch and high activation result in high forces over long MTU lengths (Hegyi et al. 2019; Kellis and Blazevich 2022). Huygaerts et al. (2020) proposed that intermuscular variations in hamstring activation could lead to disproportionate activation of one muscle and increase injury risk.

While differences in activation on an intermuscular level may explain why certain muscles are more frequently injured than their agonists, they do not account for the consistent localization of injuries within specific muscles, such as the hamstrings and the proximal BFlh (Crema et al. 2016). Uneven load distribution within the MTU has been linked to increased injury risk and may contribute to localized overload (Hedayatpour and Falla 2012; Opar et al. 2012). Sarcomere length non-uniformities occurring with muscle activation, observed in animal studies cause force variations of up to 100% of maximal isometric force (Moo et al. 2017; Li et al. 2024). This could increase injury risk as sarcomeres in series operate on different regions of the force–length curve, with some reaching near-maximal force. Moreover, considering sarcomeres in parallel muscle fibers belonging to different motor units, different activation levels across the muscle fibers could increase local non-uniformities. Here, load distribution would be uneven relative to maximal force at the operating length, rather than absolute load.

Structural and neuromuscular differences between the proximal and distal regions of the BFlh suggest that these regions have different functional roles and could further respond differently to the same contraction circumstances (Kellis 2010; Bourne et al. 2017; Fernandez-Gonzalo et al. 2016; Hegyi et al. 2018; Cerone et al. 2023). For instance, Cerone et al. observed a shift toward the proximal region in muscle activity during maximal sprinting compared to submaximal sprinting, suggesting that contraction demands may influence regional activity patterns in the BFlh. Additionally, the recruitment of motor units changes with force levels. At low force levels, predominantly slow-twitch motor units are activated, while higher force levels require the recruitment of fast-twitch motor units (Henneman, Somjen and Carpenter 1965). This may affect the amplitude and/or the spatial homogeneity of muscle activity, and could result in regionally distinct activity patterns in muscles with regional differences in fiber architecture, such as the BFlh (Kellis et al. 2010). Rahemi et al. (2014) demonstrated in a simulation study that regionalized muscle activation can alter the magnitude and direction of force transmission and possibly impact injury risk.

Furthermore, the described intramuscular variations in muscle activity can possibly lead to areas of high and areas of low activity. In a hypothetical purely serial contractile element model, force remains uniform, but low activation regions experience greater elongation. In a parallel model, length change is uniform, but high activation increases force. Both scenarios could locally increase injury risk in dynamic contractions. While this is a hypothetical mechanistic view and skeletal muscle includes contractile elements in series and in parallel, and passive structures such as Titin play a role (Herzog 2018), this hypothetical view could help in understanding the occurrence of muscle injuries.

Accordingly, this study aims to examine proximal–distal differences in regional homogeneity and amplitude of muscle activity of the BFlh, as well as the regional spatial homogeneity and amplitude of muscle activity within the proximal and distal BFlh at different knee flexion torque levels and MTU lengths. Understanding proximal–distal activation differences and regional activation patterns could help in understanding the occurrence BFlh injuries. The hypotheses are that spatial homogeneity of muscle activity in the BFlh is lower in the more frequently injured proximal region compared to the distal region and that within-region spatial homogeneity differs with isometric contractions at different knee flexion torques; that the proximal and distal regions differ in activity amplitudes; that activity amplitude decreases in both regions as MTU length increases.

## Methods

### Experimental design and participants

The study was conducted as a cross-sectional experiment including the systematic variation of the three independent variables: (1) muscle region; (2) MTU length; (3) knee flexion torque level. To determine the effect of these interventions on muscle activity and its distribution, the dependent variables were normalized amplitude of muscle activity and modified entropy (ModEn) of muscle activity (Farina et al. 2008). Amplitude of muscle activity is a measure for the extent to which the muscle fibers are activated during a contraction, and entropy, in this case, reflects how homogenously this activity is distributed over the muscles surface covered by high-density surface electromyography (HDsEMG) electrodes.

Fifteen male recreational athletes were recruited from the universities staff and students (mean age: 28.1 ± 3.4 years). Only athletes who participate at least twice a week in any kind of recreational sport were chosen to participate. The exclusion criteria were: (1) the subject having a lower extremity injury within the past year; (2) presence of neurological pathologies. Only male subjects were included to avoid the potential confounding effects of hormonal fluctuations on neuromuscular properties.

Experimental protocols and procedures were approved by the Ethics Commission of the German Sport University (169/2024) and conform to the standards set by the Declaration of Helsinki. All participants provided written informed consent after receiving a detailed explanation of the study.

### Subject preparation and electrode placement

Before conducting the measurements subjects were asked for their medical history within the past year, and notable injuries of the lower extremities in general. Subjects lay prone on a bench for electrode placement and skin preparation. The origin (tuber ischiadicum) and insertion (fibula head) of the BFlh were marked, corresponding to 0% and 100% of the MTU length, respectively. Marks for electrode grids were placed at 35% (proximal region) and 65% (distal region) along the MTU length, resulting in the proximal and distal limits of the grids being placed at approximately 25% and 75% of muscle belly length respectively. Medio-lateral placement was guided by manual palpation by an experienced physical therapist (AF). The skin within and around the marked electrode grid area was shaved, cleaned, and treated with abrasive paste to reduce impedance. Two 32-electrode HDsEMG grids (8 × 4 arrays, ~ 7.5 cm × 3.5 cm; GR10MM0804, OT Bioeletronica, Torino, Italy) were prepared with adhesive foam layers filled with electroconductive cream (AC Cream SPES Medica, Genoa, Italy). The grids were aligned with the marks and secured with elastic bands around the thigh. A reference electrode was placed on the ipsilateral ankle. The electrode grid placement used in this study was selected in accordance with An et al. (2010) and Watanabe et al. (2016) and BFlh muscle borders were palpated by the same experienced physical therapist for all subjects to avoid placing parts of the electrode grids on adjacent muscles.

### Test protocol

**Fig. 1:**
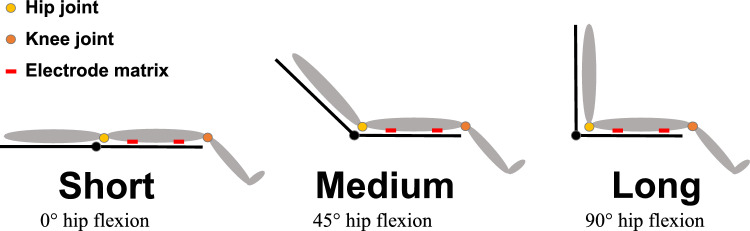
Subject positioning on the dynamometer at short (0° hip flexion angle), medium (45°), and long (90°) biceps femoris muscle-tendon unit (MTU) length. For all MTU lengths and knee flexion torque levels (30%, 60%, and 90% of the MVC90 assessed at short MTU length) knee joint angle was kept constant at 45° to control MTU lengths. To not alter the internal lever arm of the M. Biceps femoris long head about the knee joint, the knee joint angle configuration remained unchanged

All measurements were conducted on the dominant leg (preferred kicking leg) in a single session. After applying the HDsEMG electrode grids, subjects lay supine on the Isomed2000 dynamometer (D. & R. Ferstl, Hemau, Germany) in the short MTU length position (Fig. 1). The dynamometer axis was aligned with the knee's rotational axis, and the knee was fixed at 45° flexion for all measurements. The knee angle was kept constant to not alter the internal lever arm of the BFlh enable the comparison of constant knee flexion torques across MTU lengths.

The warm-up consisted of three isometric contractions at 25%, 50%, and 75% each of *self-assessed* maximal contraction intensity, held for 10 seconds. Subjects then performed a maximal isometric knee flexion contraction in the short MTU length position with verbal encouragement. A second trial at 90% of the peak torque achieved during this contraction was used as the maximum voluntary contraction (MVC90), during which subjects held the specified torque for 3 s. Data from this 3-second window were used to normalize HDsEMG and torque data, as detailed in the “Data Analysis” section.

To investigate BFlh activity at different MTU lengths and contraction intensities, subjects performed isometric knee flexion contractions at 30%, 60%, and 90% MVC90 in short, medium, and long MTU length positions (short – 0° hip flexion, medium – 45° hip flexion, long – 90° hip flexion; Fig. 1). Note that for all MTU lengths, the %MVC90 was assessed at the short MTU position for each subject, to gain a reference to ensure the same muscular output across MTU positions (i.e., constant joint torques across MTU lengths). The knee joint torque was kept constant instead of recruitment to gain insights into the regional differences in amplitude with different constant torque levels (30%_MVC90_, 60%_MVC90_, and 90%_MVC90_) across MTU lengths. Figure 1 illustrates the corresponding subject positioning for each MTU length. A total of nine trials were recorded, representing each combination of MTU length and torque level (3 MTU lengths × 3 torque levels). The order of trials was randomized. MTU lengths were randomized first, with all torque levels within a given MTU length conducted consecutively to minimize movement and maintain good electrode-to-skin contact. Within each MTU length, the order of torque levels was also randomized. Hip angles were controlled using an electronic goniometer on the Isomed2000 and verified with a manual goniometer. For each trial, subjects used a ramp-up speed of 30%_MVC90_ per second to reach the target torque level (30%_MVC90_, 60% _MVC90_, or 90% _MVC90_) and held the specified knee flexion torque for 10 seconds. Since subjects only had a short familiarization period with holding target torques, 10 seconds were chosen to increase the likelihood of stable torque plateaus. Individual trials were separated by 1 minute of rest. Torque levels were based on the MVC90 measurement performed in the short MTU length and kept constant across all MTU lengths. This ensured that subjects reached the same absolute torque for each intensity (30%_MVC90_, 60% _MVC90_, or 90% _MVC90_) regardless of MTU length. Normalizing torque to the short MTU length allowed for analysis of regional BFlh behavior along the muscle's force-length curve due to consistent knee flexion torque across MTU lengths.

## Data collection and processing

### Data collection

HDsEMG data were recorded with the aforementioned HDsEMG electrodes, sampled in monopolar detection mode and digitized by a 16-bit A/D amplifier (Sessantaquattro, OT Bioelettronica, Torino, Italy) with a sampling rate of 2000 Hz. The HDsEMG data were online Bandpass filtered (10.5–500 Hz). Knee flexion torque data were recorded with the Isomed2000, sampled at 2000 Hz and synced to the HDsEMG data via the 16-bit A/D amplifier.

### Data processing

Data processing was conducted offline using Matlab (Matlab R2023b, MathWorks, Inc., Natick, USA). HDsEMG data were filtered using a fourth-order Butterworth filter with cut-offs at 15 and 350 Hz to isolate muscle activation patterns (Jordanic et al. 2017). Torque data were filtered with a fourth-order low-pass Butterworth filter at 20 Hz and smoothed using a 500 ms moving average without overlap. The quality of each HDsEMG channel was assessed visually and automatically for outliers (Malešević et al. 2021) and percent residual difference (Farago and Chan 2023). Channels with insufficient signal quality were interpolated using 2D spline interpolation with neighboring channels. Outliers were calculated according to Malešević et al. 2021 and detected Outlier channels were interpolated. Percent residual difference was calculated according to Farago and Chan (2023) and the threshold (thPRD) for insufficient quality channels was set as thPRD = min{median(PRDc) + τ, median(PRDc) + Φstd(PRDc)}, as it was done by the original authors of the paper. τ and Φ are tunable constants (Φ standard deviations greater than the median PRD of all channels), and τ is used to reduce false positives (fixed min. threshold). τ and Φ were set to 50 and 6, respectively, as was done by Farago and Chan. Of the in total 9600 recorded signals, 31 (0.3%) were interpolated. Afterward, the data were full-wave rectified, converted to single differential format (algebraic difference between two adjacent electrodes in fiber direction), and smoothed using root-mean-square (RMS) with a 500 ms window without overlap. As single differential conversion was conducted in fiber direction along electrode columns, the monopolar grid layout of 8 × 4 (32 monopolar signals) changed to a 7 × 4 grid of 28 single differential signals.

Normalization of HDsEMG data was performed by identifying the 1-s window with the lowest variability during which torque exceeded the 95th percentile of the trial. The RMS values from this period were averaged and used as the MVC90 activity reference. All RMS values for trials and channels were normalized to this MVC90 activity. Torque data were similarly averaged over this period and normalized to the short MTU length MVC90.

**Fig. 2:**
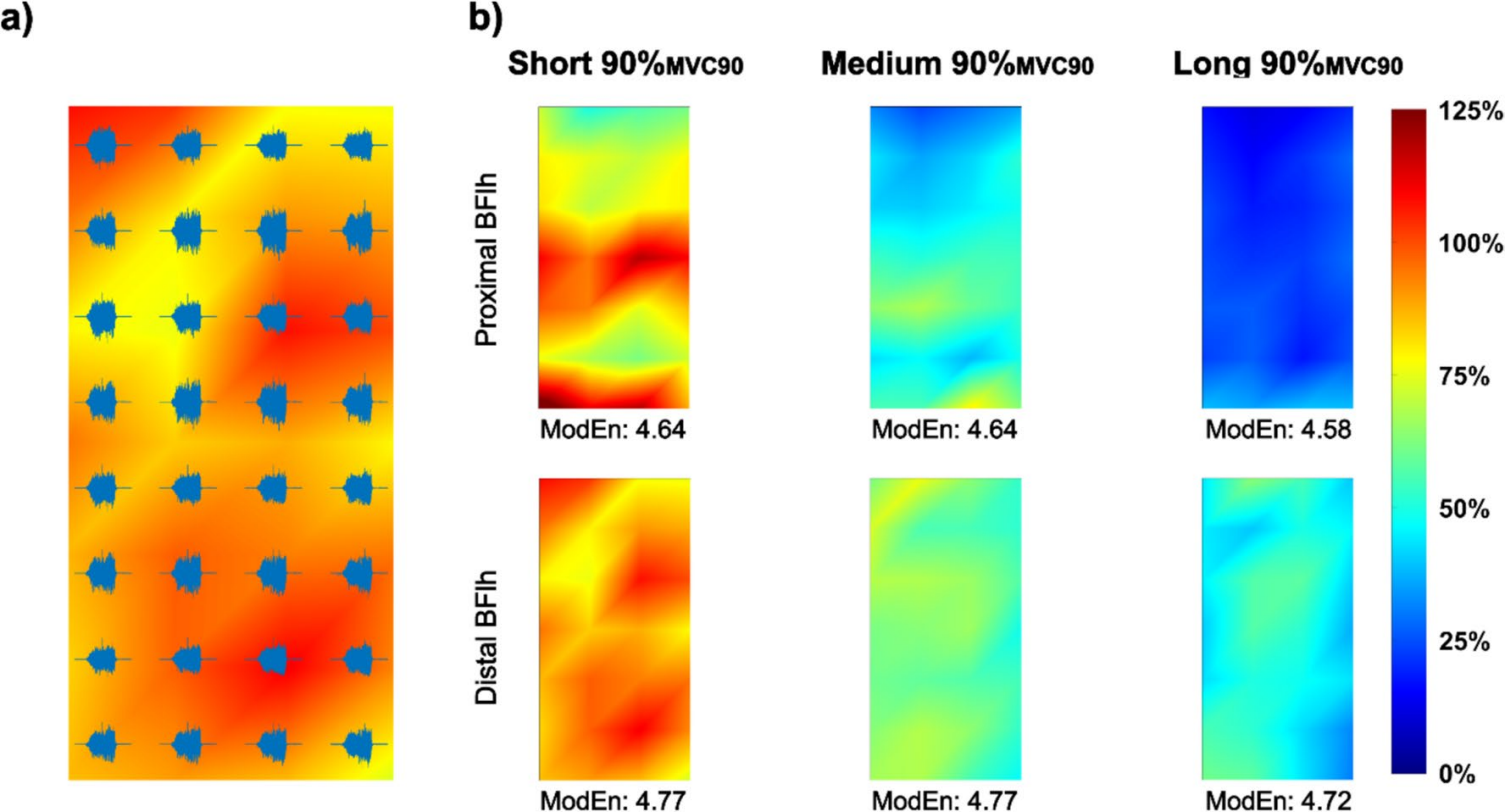
**a** Example of the 32 raw HDsEMG monopolar signals from the distal region of the Biceps femoris long head (BFlh) overlaid on a monopolar activation map, illustrating the relationship between the spatial arrangement of the electrode matrix and the corresponding activity patterns. **b** Activation maps visualizing the data of 28 single differential HDsEMG signals via normalized muscle activity (%_MVC90_) for the proximal and distal regions of the BFlh during 90%_MVC90_ contractions at short, medium, and long MTU lengths. Warmer colors (red) indicate higher, and cooler colors (blue) indicate lower activity amplitudes. Modified entropy (ModEn) values are displayed below each activation map, with higher values indicating greater regional spatial homogeneity of muscle activation

For further analysis, a stable torque period was identified for each trial, defined as three consecutive 500 ms windows with the lowest torque variability and deviation from the target torque below 5%_MVC90_. Trials failing to meet these criteria were excluded. Activation maps were created for each subject and condition by arranging the normalized RMS data of the 1500 ms stable torque period to reflect the spatial layout of the electrode grid (Fig. 2). Solely for visualization purposes for the activation maps the 28 normalized single differential signals were used to interpolate activity between electrodes. From these maps, the mean normalized RMS value for each region was calculated to represent overall activity, and modified entropy (ModEn) was computed using the 28 normalized single differential RMS values from the stable torque period (Farina et al. 2008). ModEn values range from 0 (completely heterogeneous activation) to log2 of the number of signals (maximum homogeneity). The maximum ModEn value in this study was 4.807 (completely homogenous activity).

## Statistical analysis

A three-way repeated measures ANOVA was conducted to examine the effects of muscle region (proximal and distal), MTU length (short, medium, and long), and knee flexion torque level (30%_MVC90_, 60%_MVC90_, and 90%_MVC90_) on the dependent variables of RMS amplitude and ModEn of muscle activity. Each factor was treated as a within-subject variable, and the Greenhouse–Geisser correction was applied to adjust for any violations of the assumption of sphericity. Effect sizes are reported using partial eta-squared (η^2^p) to quantify the magnitude of observed effects from ANOVA results and Cohen’s d is reported for post hoc comparisons. Statistical significance was set at an alpha level of 0.05.

If a significant three-way interaction was found, simple two-way interactions were conducted at each level of the third factor, with Bonferroni corrections applied. Significant simple two-way interactions were followed by simple main effects, which were further analyzed with simple pairwise comparisons using Holm’s correction (1979). If the three-way interaction was not significant, two-way interactions were analyzed directly from the ANOVA output. Significant two-way interactions were then followed by simple main effects and simple pairwise comparisons, with *p* values corrected according to Holm.

## Results

### Effects of torque and MTU length on amplitude of muscle activity

**Fig. 3:**
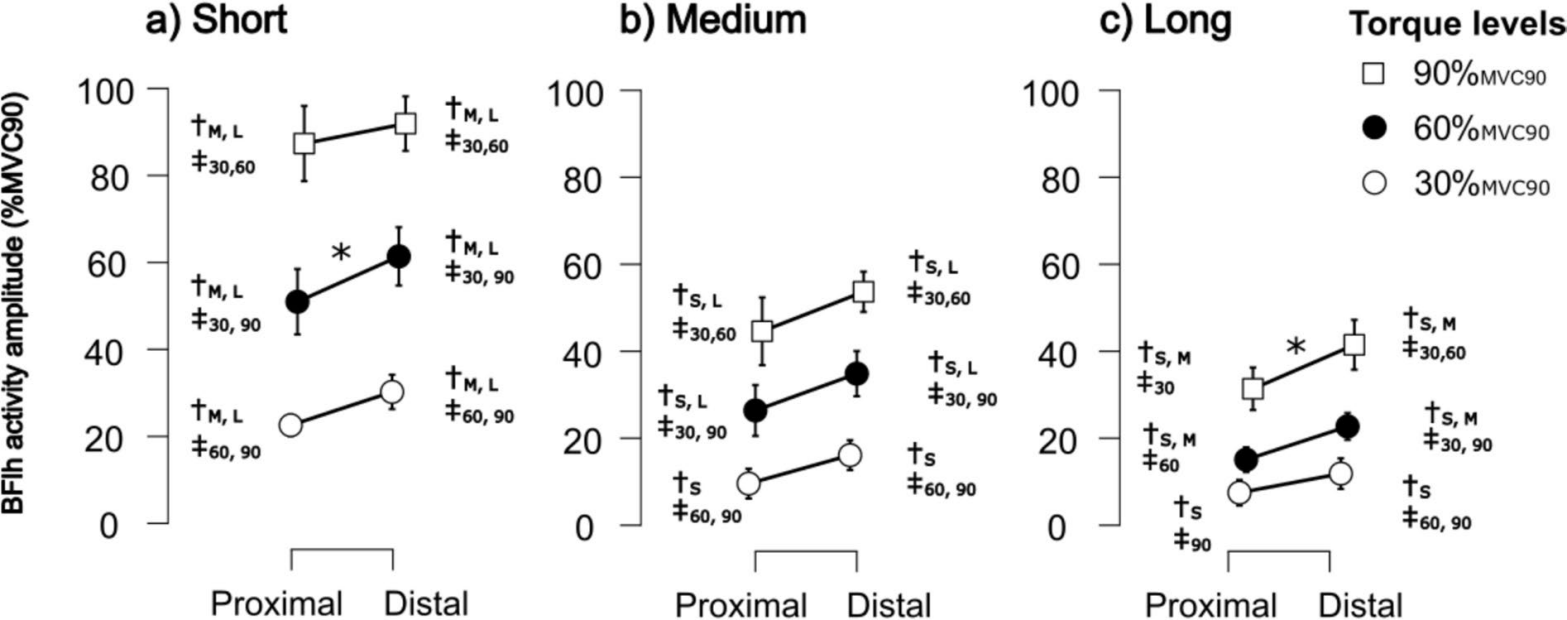
Normalized muscle activity amplitude (%_MVC90_) of the proximal and distal regions of the Biceps femoris long head (BFlh) at different MTU lengths and torque levels. **a** Short MTU length (S), **b** Medium MTU length (M), **c** Long MTU length (L). Data are shown for three torque levels: 30%_MVC90_ (black circles), 60%_MVC90_ (gray circles), and 90%_MVC90_ (white squares). Significant differences between MTU lengths at the same torque level are indicated by †, and significant differences between torque levels at the same MTU length are denoted by ‡. Asterisks (*) indicate significant proximal–distal differences (*p* < 0.05). Error bars represent the standard deviation

The analysis revealed that there was a statistically significant three-way interaction between the effects of muscle region, length, and torque level on activity amplitude (F(4, 36) = 3.979, *p* = 0.009, η^2^p = 0.307). Individual activity maps of one subject are shown in Fig. 2 and mean activity amplitudes for each condition and region are shown in Fig. 3.

The factors Region ✻ Torque showed a significant interaction in the short MTU length (*F*(2,24) = 3.775, *p* = 0.038, η^2^p = 0.239), but not in the medium or long MTU lengths. For Region ✻ Length there were no significant interactions at any torque level. Simple main effects for the significant Region ✻ Torque interaction in the short MTU length revealed significantly lower amplitude of muscle activity between in the proximal region than in the distal regions at 30%_MVC90_ (*F*(1,12) = 8.420, *p* = 0.013) and 60%_MVC90_ *F*(1,12) = 21.899, *p* < 0.001) but not at 90%_MVC90_ (*F*(1,12) = 2.121, *p* = 0.171).

For both the proximal and distal region, the interaction between Length ✻ Torque was statistically significant for the amplitude of muscle activity (Proximal: *F*(4,40) = 28.486, *p* < 0.001; Distal: *F*(4,40) = 25.566, *p* < 0.001). Simple main effect analyses for this interaction revealed a statistically significant increase of activity amplitude with increasing torque at each length for both regions, and statistically significant decrease of amplitude with increasing length at each torque level in both regions (Table 1).

**Table 1:** Post hoc results of the simple main effects of torque on amplitude at each MTU length and the effects of MTU length on amplitude at each torque level

	Sum of squares	df	Mean square	*F*	*p*
*Simple main effects of knee flexion torque on amplitude at each MTU length*					
* Proximal*					
S	2.128	2	1.064	126.981	< 0.001
M	0.623	2	0.311	56.938	< 0.001
L	0.245	2	0.121	20.799	< 0.001
* Distal*					
S	1.903	2	0.952	161.92	< 0.001
M	0.706	2	0.353	140.482	< 0.001
L	0.449	2	0.225	97.018	< 0.001
*Simple main effects of MTU length on amplitude at each knee flexion torque level*					
* Proximal*					
30%_MVC90_	0.163	2	0.081	64.658	< 0.001
60%_MVC90_	0.66	2	0.33	48.279	< 0.001
90%_MVC90_	1.918	2	0.959	125.894	< 0.001
* Distal*					
30%_MVC90_	0.185	2	0.092	71.2	< 0.001
60%_MVC90_	0.783	2	0.391	77.046	< 0.001
90%_MVC90_	1.385	2	0.693	198.047	< 0.001

Degrees of freedom (df), *F* value (*F*), *p* value (*p*)

90% of maximum voluntary contraction knee flexion torque (MVC90)

In the proximal region significantly lower amplitude was observed than in the distal region at short- 60%_MVC90_ (mean difference = − 0.104, SE = 0.025, *t* = − 4.099, Cohen’s d = − 1.193, P_Holm_ = 0.024) and long- 90%_MVC90_ (mean difference = − 0.101, SE = 0.025, *t* = − 3.971, Cohen’s d = − 1.155, P_Holm_ = 0.031). Furthermore, comparing different conditions within one region showed significant differences in all comparisons, except when comparing medium- 30%_MVC90_ to long- 30%_MVC90_ in both regions, and when comparing long- 30%_MVC90_ to long- 60%_MVC90_ in the proximal region, with amplitude increasing with increasing torque and decreasing with increasing length in both BFlh regions (Fig. 3, see table in appendix for detailed statistical results).

### Effects of torque and MTU length on spatial homogeneity of muscle activity (modEn)

**Fig. 4:**
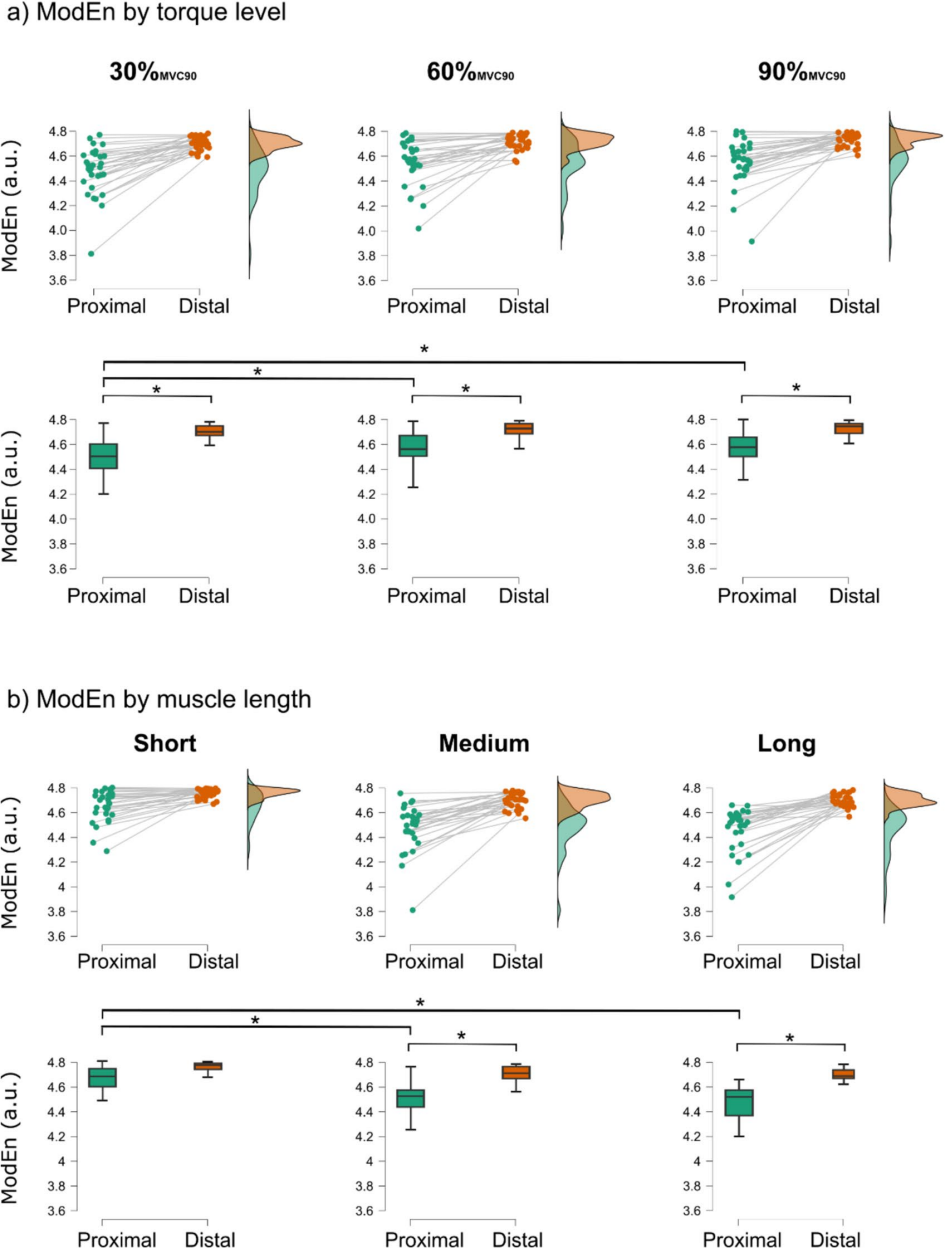
Individual modified entropy (ModEn) values for each subject and condition in the proximal (green) and distal (orange) regions of the Biceps femoris long head (BFlh). Density plots on the right show the distribution of ModEn values by region. Boxplots below illustrate the same data, with high ModEn values representing homogenous activation and low values indicating heterogeneous activation. **a** ModEn as a function of torque level (30%, 60%, and 90%_MVC90_). **b** ModEn as a function of MTU length (short, medium, and long). Asterisks denote statistically significant differences (*p* < 0.05)

The three-way interaction between the factors torque, MTU length, and region was not statistically significant (F(4,36) = 0.323, p = 0.861). However, statistically significant two-way interactions were observed between the factors Region ✻ Length (*F*(2,18) = 5.588, *p* = 0.013, η^2^_p_ = 0.383) and Region ✻ Torque (*F*(2,18) = 5.088, *p* = 0.018, η^2^_p_ = 0.361). Mean ModEn values by length and by torque are reported in Fig. 4.

Simple main effect analyses revealed proximal–distal differences at all torque levels (30%_MVC90_ – *F*(1,12) = 48.688, *p* < 0.001; 60%_MVC90_ – *F*(1,12) = 17.150, *p* = 0.003; 90%_MVC90_ – *F*(1,12) = 23.955, *p* < 0.001). These comparisons showed lower spatial homogeneity in the proximal region with strong effects across all torque levels, with the largest effect size observed at 30%_MVC90_. Mean values are shown in Fig. 4a and statistical test results are reported in Table 2.

**Table 2:** Pairwise post hoc comparisons for spatial homogeneity of muscle activity between proximal and distal BFlh regions at each torque level (30%_MVC90_, 60%_MVC90_, and 90%_MVC90_) and MTU length (short, medium, long), during isometric knee flexion contractions. Negative mean differences indicate lower spatial homogeneity in the proximal region compared to the distal region

		Mean difference	SE	*t*	Cohen’s d	Pholm
*Torque levels*						
Proximal 30%_MVC90_	Distal 30%_MVC90_	− 0.222	0.037	− 6.026	− 1.779	0.001
Proximal 60%_MVC90_	Distal 60%_MVC90_	− 0.174	0.037	− 4.705	− 1.389	0.006
Proximal 90%_MVC90_	Distal 90%_MVC90_	− 0.177	0.037	− 4.804	− 1.418	0.006
*MTU lengths*						
Proximal S	Distal S	− 0.104	0.044	− 2.365	− 0.836	0.203
Proximal M	Distal M	− 0.222	0.044	− 5.024	− 1.777	< 0.001
Proximal L	Distal L	− 0.247	0.044	− 5.581	− 1.974	< 0.001

Standard error (SE), *t*-statistic (t), Effect size (Cohen’s d), Adjusted *p* values (Pholm)

Short MTU (S), medium MTU (M), and long MTU (L)

90% of maximum voluntary contraction knee flexion torque (MVC90)

In the proximal region, spatial homogeneity increased with increasing torque. Significant differences between 30%_MVC90_ and 60%_MVC90_ (mean difference = − 0.068, standard error (SE) = 0.014, *t* = − 4.807, Cohen’s d = − 0.541, P_Holm_ < 0.001) and 30%_MVC90_ and 90%_MVC90_ (mean difference = − 0.070, SE = 0.014, *t* = − 5.625, Cohen’s d = − 0.633, P_Holm_ < 0.001) were found, but not between 60%_MVC90_ and 90%_MVC90_. Comparing spatial homogeneity in the distal region across torque levels did not reveal any statistically significant differences and mean values were similar across torque levels (Fig. 4a).

Simple main effects analyses indicated that the proximal and distal BFlh regions differed significantly in spatial homogeneity of muscle activity at all MTU lengths (short – *F*(1,12) = 14.588, *p* = 0.004; medium – *F*(1,12) = 21.472, *p* = 0.001; long – *F*(1,12) = 21.612, *p* = 0.001). Pairwise post hoc comparison between the proximal and distal regions were not significant at the short MTU length. However, at medium and long MTU lengths significantly lower spatial homogeneity was observed in the proximal region, with higher effect sizes at the long MTU length. Mean values are shown in Fig. 4b and statistical test results reported in Table 2.

In the proximal region, a significant decrease in spatial homogeneity was found with longer lengths (short vs. medium length: mean difference = 0.174, SE = 0.035, *t* = 5.022, Cohen’s d = 1.396, P_Holm_ < 0.001; short vs. long length: mean difference = 0.198, SE = 0.035, *t* = 5.701, Cohen’s d = 1.584, P_Holm_ < 0.001). In contrast no significant effects of MTU length were observed in the distal region (Fig. 4b).

## Discussion

The aim of this study was to assess regional differences in the homogeneity of spatial distribution and amplitude of muscle activity within the BFlh, as non-uniform activation patterns may contribute to uneven load distribution and increase injury risk. The results support the hypotheses: the spatial distribution of muscle activity is less homogeneous in the more frequently injured proximal region and increases with increased knee flexion torque in the proximal region but decreases with increasing MTU length; the proximal and distal regions differ in muscle activity amplitudes; and activity amplitude decreases in both regions with increasing MTU length. These findings highlight the non-uniform activation patterns within the BFlh.

### Regional muscle activity of the BFlh

In this study, the proximal BFlh showed lower regional spatial homogeneity and amplitude than the distal region, with differences most pronounced at longer muscle lengths and lower torque levels. Additionally, activity amplitudes were consistently lower in the proximal region across conditions. While mechanical loading was not directly measured, these results suggest that lower spatial homogeneity in the proximal BFlh may contribute to uneven load distribution within the muscle. Non-uniform muscle activity could result in localized peaks of activation, potentially leading to combined active and passive tension exceeding the injury threshold of specific fibers or fascicles. Conversely, regions of low activity may lead to insufficient active tension, leaving fibers unprepared for external loads and more susceptible to passive elongation and rupture. These patterns may provide a mechanistic explanation for the higher injury incidence in the proximal BFlh during high-speed, dynamic contractions. Given that muscle force production depends on activation, length, and velocity, and that in the present study only MTU length was varied while knee flexion torque was held constant, the observed decrease in muscle activity with increasing length suggests operation on the ascending limb of the force–length relationship. This interpretation is supported by Kellis and Blazevich (2022), who reached similar conclusions regarding the hamstrings joint moment–angle relationship. However, as the measured torque represents the combined contribution of all agonist and synergist muscles, this does not provide direct evidence for the proposed interpretation.

Torque levels (30%_MVC90_, 60%_MVC90_, and 90%_MVC90_) and the internal lever arm of the BFlh (due to 45° knee flexion in all positions) were kept constant across MTU length positions. Amplitude of muscle activity was lower at longer MTU lengths in both regions (Figs. 2b, 3), consistent with the ascending limb of the force–length relationship, where longer lengths require less activation to achieve the same torque. Torque levels were normalized to MVC90 at the short MTU, influencing relative contraction intensity across lengths. This normalization likely contributed to differences in activity amplitude between short, medium, and long MTU lengths, by introducing slight variations in the electrode pick-up area between the normalization measurement and condition trials. MTU length was manipulated by varying the hip angle while keeping the knee angle fixed, isolating changes in activation patterns to MTU length without altering the internal BFlh lever at the knee. While this setup using isometric contractions minimized muscle movement under the skin for robust homogeneity calculations, it does not replicate dynamic conditions with simultaneous changes in hip and knee angles in which injuries typically occur.

Currently, no literature directly examined regional differences in regional spatial homogeneity of muscle activity within the proximal and distal BFlh, which limits direct comparisons. However, Schlink et al. (2020) reported that the BFlh exhibited the lowest spatial homogeneity among all lower-extremity muscles they examined at higher running speeds. Literature further shows that the BFlh is the most frequently injured muscle among those included in Schlink et al.’s study (Ekstrand et al. 2011), especially at higher running speeds (Chumanov et al. 2007, Duhig et al. 2016). Previous studies reported regional amplitude differences in the BFlh, with higher distal activation during running and exercise tasks (Hegyi et al. 2018, 2019), suggesting region-specific activation patterns. Only Süskens et al. (2023) observed higher normalized proximal activation. This discrepancy may partly stem from one methodological difference: Süskens et al. used conventional bipolar electromyography (EMG) focused on the most proximal electrodes, whereas Hegyi et al. assessed proximal activation using multiple HDsEMG electrodes across the proximal region. Both Süskens et al. (2023) and Cerone et al. (2023) investigated maximal running speeds while Süskens et al. found the highest activation in the proximal region, Cerone et al. reported a proximal shift in the center of activation, indicating a shift in demands for the proximal region. These results suggest that commonly used hamstring exercises may not adequately prepare the proximal BFlh for the demands of maximal sprinting.

Reduced homogeneity in the proximal region during high-speed movements may exacerbate localized variations in active tension, contributing to its susceptibility to injury. Hegyi et al. (2018, 2019) similarly suggested that step-by-step variations in activation patterns could increase injury risk, particularly under dynamic conditions. Collectively, these findings suggest an important role of spatial activation patterns in conjunction with mechanical demands in influencing injury risk in the proximal BFlh. While the absolute differences in modified entropy were modest, individual ModEn values showed up to 20% variance and may reflect meaningful changes in the spatial distribution of activation. Whether such differences contribute directly to increased injury risk remains unclear and future studies using fatiguing or dynamic contractions could help determine their functional relevance.

### Proximal and distal BFlh response to changing torque levels and MTU lengths

The present findings highlight that the proximal BFlh shows greater variability in regional spatial homogeneity across torque levels and MTU lengths compared to the distal region. Specifically, regional spatial homogeneity in the proximal region increased with higher torque levels but decreased with longer MTU lengths. This contrasts with the distal region, which maintained consistent activation patterns across all conditions. The need for the proximal region to adjust its activation patterns to varying contraction circumstances may indicate a lack of a consistent general activation strategy. If true, this likely increases the occurrence of step-by-step differences in muscle activity amplitude and spatial distribution, contributing to uneven load distribution and increasing the risk of localized overloading during dynamic movements, as hypothesized by Hegyi et al. (2018, 2019). Both regions seem to operate on the ascending limb of the force–length relationship in this setup, as activity amplitude decreased with increasing MTU length.

Possible functional distinctions between the proximal and distal BFlh remain unclear, but the observed differences in activation patterns coupled with regional architectural differences (Kellis, 2010) could reflect potential regional specialization.

## Limitations

The presented study has some limitations related to the influence of the location of the innervation zone in relation to the electrode grids. Although isometric contractions minimize variations in the electrode pick-up area, with MTU length changes muscle displacement under the skin is inevitable and results in slightly different pick-up areas of each electrode grid and it might have resulted in shifts of the innervation zone in different MTU lengths in relation to the electrode locations. Additionally, the normalization procedure—based on maximal contractions at the shortest length—may have introduced slight inconsistencies when normalizing data from longer MTU lengths to the MVC measured in the shortest MTU length. However, the consistent ModEn trends across all MTU lengths and the alignment of normalized amplitude patterns observed in our study and in the literature suggest that the effects are physiological rather than methodological. If ModEn changes were primarily due to the innervation zone shifting in or out of the grid, we would expect a marked variation in proximal and distal ModEn with MTU length. Instead, distal ModEn remained stable, while proximal ModEn consistently decreased in homogeneity with higher torque and shorter lengths. This pattern supports a physiological origin of the ModEn differences. Furthermore, cross-talk from neighboring muscles was minimized by careful electrode placement and the usage of small electrodes (10 mm diameter). Nevertheless, with small margins between electrode grids and muscle borders some residual interference from neighboring muscles cannot be fully excluded. Lastly, our highly controlled single-joint isometric conditions do not replicate the dynamic, high-strain contractions under which BFlh injuries typically occur. However, this study demonstrated that even under isometric conditions, MTU length and force level influenced activation homogeneity, suggesting that inhomogeneous activation may be even more pronounced during eccentric contractions.

## Practical implications

In light of the literature, the findings suggest that current prevention exercises, like the Nordic Hamstring Exercise, may insufficiently activate the proximal BFlh (Hegyi et al. 2018), potentially leaving it under-prepared for high-speed sprinting. As high-intensity contractions in the present study showed higher regional spatial homogeneity of muscle activity, high-intensity training, particularly under maximal torque and varying MTU lengths, could improve spatial homogeneity in the proximal region and possibly reduce its vulnerability to injury.

For future research, investigating whether training can alter regional spatial homogeneity and how different contraction modes, particularly eccentric contractions, affect homogeneity is important. Additionally, exploring the effects of fatigue on spatial homogeneity is critical, as fatigue could be an important factor in BFlh injuries (Huygaerts et al. 2020). Understanding how muscle injury impacts regional spatial homogeneity, could provide valuable insights into designing effective rehabilitation strategies and preventing re-injury.

## Conclusion

The proximal BFlh exhibits lower regional spatial homogeneity and amplitude of muscle activity compared to the distal region, reflecting non-uniform activation patterns that may contribute to its higher susceptibility to injury, particularly during high-speed or high-load activities. Additionally, the proximal region also showed greater variability in activation patterns across torque levels and MTU lengths, suggesting a need for greater changes in activation under changing conditions. Such greater changes in activation under varying conditions could be problematic during high-speed sprinting, where rapid and repeated shifts in mechanical demand occur throughout the running cycle. These findings could suggest that the proximal region’s lower activity and homogeneity may impair its ability to manage mechanical demands consistently under changing contraction conditions and thus contribute to injury risk.

However, these findings were derived from isometric conditions, and it remains unclear whether similar patterns persist during dynamic actions, such as sprinting, where injury mechanisms are more complex. Future research should examine the influence of contraction circumstances and mode, such as high voluntary muscle activation at long MTU lengths or repetitive eccentric contractions, fatigue, and prior injuries on muscle activation patterns and consider whether targeted interventions could enhance regional homogeneity of activation and possibly reduce injury risk in the proximal BFlh.

## Data Availability

ModEn and normalized amplitude values, which were used for the reported statistics in this paper, are available on figshare (10.6084/m9.figshare.28060874). The raw HDsEMG data are not publicly available but can be obtained from the authors upon reasonable request.

## Appendix

**Table:**

Length comparisons		Mean difference	SE	*t*	Cohen’s d	Pholm	
*Proximal region*							
Proximal, S, 30	Proximal, M, 30	0.13	0.029	4.548	1.485	0.002	**
Proximal, S, 30	Proximal, L, 30	0.151	0.029	5.279	1.724	< 0.001	***
Proximal, M, 30	Proximal, L, 30	0.021	0.029	0.732	0.239	1	
Proximal, S, 60	Proximal, M, 60	0.246	0.029	8.597	2.807	< 0.001	***
Proximal, S, 60	Proximal, L, 60	0.359	0.029	12.555	4.1	< 0.001	***
Proximal, M, 60	Proximal, L, 60	0.113	0.029	3.958	1.292	0.01	**
Proximal, S, 90	Proximal, M, 90	0.428	0.029	14.956	4.884	< 0.001	***
Proximal, S, 90	Proximal, L, 90	0.56	0.029	19.582	6.395	< 0.001	***
Proximal, M, 90	Proximal, L, 90	0.132	0.029	4.626	1.511	0.001	**
*Distal region*							
Distal, S, 30	Distal, M, 30	0.141	0.029	4.938	1.613	< 0.001	***
Distal, S, 30	Distal, L, 30	0.184	0.029	6.424	2.098	< 0.001	***
Distal, M, 30	Distal, L, 30	0.042	0.029	1.486	0.485	1	
Distal, S, 60	Distal, M, 60	0.265	0.029	9.288	3.033	< 0.001	***
Distal, S, 60	Distal, L, 60	0.387	0.029	13.533	4.419	< 0.001	***
Distal, M, 60	Distal, L, 60	0.121	0.029	4.245	1.386	0.004	**
Distal, S, 90	Distal, M, 90	0.382	0.029	13.38	4.37	< 0.001	***
Distal, S, 90	Distal, L, 90	0.504	0.029	17.646	5.762	< 0.001	***
Distal, M, 90	Distal, L, 90	0.122	0.029	4.265	1.393	0.004	**
Torque comparisons							
*Proximal region*							
Proximal, S, 30	Proximal, S, 60	− 0.284	0.028	− 9.968	− 3.244	< 0.001	***
Proximal, S, 30	Proximal, S, 90	− 0.648	0.028	− 22.742	− 7.401	< 0.001	***
Proximal, S, 60	Proximal, S, 90	− 0.364	0.028	− 12.775	− 4.157	< 0.001	***
Proximal, M, 30	Proximal, M, 60	− 0.168	0.028	− 5.904	− 1.921	< 0.001	***
Proximal, M, 30	Proximal, M, 90	− 0.35	0.028	− 12.297	− 4.002	< 0.001	***
Proximal, M, 60	Proximal, M, 90	− 0.182	0.028	− 6.393	− 2.08	< 0.001	***
Proximal, L, 30	Proximal, L, 60	− 0.076	0.028	− 2.667	− 0.868	0.286	
Proximal, L, 30	Proximal, L, 90	− 0.239	0.028	− 8.389	− 2.73	< 0.001	***
Proximal, L, 60	Proximal, L, 90	− 0.163	0.028	− 5.722	− 1.862	< 0.001	***
*Distal region*							
Distal, S, 30	Distal, S, 60	− 0.312	0.028	− 10.944	− 3.561	< 0.001	***
Distal, S, 30	Distal, S, 90	− 0.617	0.028	− 21.66	− 7.049	< 0.001	***
Distal, S, 60	Distal, S, 90	− 0.305	0.028	− 10.717	− 3.487	< 0.001	***
Distal, M, 30	Distal, M, 60	− 0.187	0.028	− 6.579	− 2.141	< 0.001	***
Distal, M, 30	Distal, M, 90	− 0.376	0.028	− 13.188	− 4.292	< 0.001	***
Distal, M, 60	Distal, M, 90	− 0.188	0.028	− 6.61	− 2.151	< 0.001	***
Distal, L, 30	Distal, L, 60	− 0.109	0.028	− 3.81	− 1.24	0.013	*
Distal, L, 30	Distal, L, 90	− 0.296	0.028	− 10.399	− 3.384	< 0.001	***
Distal, L, 60	Distal, L, 90	− 0.188	0.028	− 6.589	− 2.144	< 0.001	***

All p values adjusted for comparing a family of 153; ^*^*p* < 0.05, ^**^*p* < 0.01, ****p* < 0.001

## Abbreviations

ANOVA: Analysis of variance
BFlh: Biceps femoris long head
df: Degrees of freedom
EMG: Electromyography
HDsEMG: High-density surface electromyography
ModEn: Modified entropy
MTU: Muscle-tendon unit
MVC: Maximum voluntary contraction
MVC90: 90% knee flexion of maximum voluntary contraction knee flexion torque
RMS: Root mean square
SE: Standard error
